# Age-group associations of schistosomiasis prevalence from trial data, Côte d’Ivoire, Kenya and the United Republic of Tanzania

**DOI:** 10.2471/BLT.23.289843

**Published:** 2024-02-29

**Authors:** Ryan E Wiegand, Maurice R Odiere, Safari Kinung’hi, Eliézer K N'Goran, Pauline Mwinzi, W Evan Secor

**Affiliations:** a Division of Parasitic Diseases and Malaria, Centers for Disease Control and Prevention, 1600 Clifton Road NE, MS H24-5, Atlanta, Georgia, GA 30329, United States of America.; b Centre for Global Health Research, Kenya Medical Research Institute, Kisumu, Kenya.; c National Institute for Medical Research, Mwanza Centre, Mwanza, United Republic of Tanzania.; d Université Félix Houphouët-Boigny, Abidjan, Côte d'Ivoire.; e Expanded Special Programme for Elimination of Neglected Tropical Diseases, World Health Organization Regional Office for Africa, Brazzaville, Congo.

## Abstract

**Objective:**

To determine if the prevalence of schistosomiasis in children aged 9–12 years is associated with the prevalence in 5–8-year-olds and adults after preventive chemotherapy in schools or the community.

**Methods:**

We combined data from four community-randomized, preventive chemotherapy trials in treatment-naïve populations in Côte d’Ivoire, Kenya and the United Republic of Tanzania during 2010–2016 according to the number of praziquantel treatments and the delivery method. *Schistosoma mansoni* infection was sought on two slides prepared from each participant’s first stool using the Kato–Katz technique. We assessed associations between *S. mansoni* prevalence in 9–12-year-olds and 5–8-year-olds and adults in the community before and after treatment using Bayesian regression models.

**Findings:**

Stool samples from 47 985 5–8-year-olds, 81 077 9–12-year-olds and 20 492 adults were analysed. We found associations between the prevalence in 9–12-year-olds and that in 5–8-year-olds and adults after preventive treatment, even when only school-age children were treated. When the prevalence in 9–12-year-olds was under 10%, the prevalence in 5–8-year-olds was consistently under 10%. When the prevalence in 9–12-year-olds was under 50%, the prevalence in adults after two or four rounds of preventive chemotherapy was 10%–15% lower than before chemotherapy. Post-chemotherapy age-group associations were consistent with pre-chemotherapy associations in this analysis and previous studies.

**Conclusion:**

The prevalence of *S. mansoni* infection in 9–12-year-olds was associated with the prevalence in other age groups and could be used to guide community treatment decisions.

## Résumé

**Objectif:**

Déterminer si la prévalence de la schistosomiase chez les enfants de 9 à 12 ans est associée à celle observée chez les 5–8 ans et les adultes après une chimiothérapie préventive au sein des écoles ou de la communauté.

**Méthodes:**

Nous avons regroupé les données provenant de quatre essais randomisés de chimiothérapie préventive menés en milieu communautaire auprès de populations n'ayant jamais été traitées en Côte d'Ivoire, au Kenya et en République-Unie de Tanzanie entre 2010 et 2016, en fonction du nombre de traitements au praziquantel et du mode d'administration. Une infection à *Schistosoma mansoni* a été recherchée sur deux lames préparées à partir des premières selles de chaque participant, à l'aide de la technique de Kato-Katz. Nous avons employé des modèles de régression bayésienne qui nous ont permis d'identifier un rapport entre la prévalence de *S. mansoni* au sein de la communauté chez les 9–12 ans d'une part, et chez les 5–8 ans et les adultes de l'autre, avant et après traitement.

**Résultats:**

Les échantillons de selles de 47 985 enfants âgés de 5 à 8 ans, 81 077 enfants de 9 à 12 ans et 20 492 adultes ont été analysés. Nous avons constaté qu'après un traitement préventif, la prévalence chez les 9–12 ans ainsi que chez les 5–8 ans et les adultes était liée, même quand seuls les enfants d'âge scolaire étaient traités. Lorsque la prévalence chez les 9–12 ans était inférieure à 10%, celle chez les 5–8 ans était systématiquement sous les 10%. Lorsque la prévalence chez les 9–12 ans était inférieure à 50%, celle chez les adultes après deux ou quatre traitements par chimiothérapie préventive était 10 à 15% plus faible qu'avant toute chimiothérapie. Les liens par catégorie d'âge en post-chimiothérapie étaient cohérents avec ceux en pré-chimiothérapie, tant dans la présente analyse que dans de précédentes études.

**Conclusion:**

La prévalence de l'infection à *S. mansoni* chez les 9–12 ans était associée à la prévalence dans d'autres catégories d'âge, ce qui pourrait servir à orienter les décisions de prise en charge au sein de la communauté.

## Resumen

**Objetivo:**

Determinar si la prevalencia de la esquistosomiasis en niños de 9 a 12 años está asociada con la prevalencia en niños de 5 a 8 años y adultos tras la quimioterapia preventiva en escuelas o en la comunidad.

**Métodos:**

Se combinaron datos de cuatro ensayos aleatorizados a nivel comunitario de quimioterapia preventiva en poblaciones sin tratamiento en Costa de Marfil, Kenia y la República Unida de Tanzania entre 2010 y 2016 según el número de tratamientos con praziquantel y el método de administración. La infección por *Schistosoma mansoni* se buscó en dos laminillas preparadas a partir de las primeras heces de cada participante mediante la técnica de Kato-Katz. Se evaluaron las asociaciones entre la prevalencia de *S.* *mansoni* en niños de 9 a 12 años y de 5 a 8 años y adultos en la comunidad antes y después del tratamiento mediante modelos de regresión bayesiana.

**Resultados:**

Se analizaron muestras de heces de 47 985 niños de 5 a 8 años, 81 077 niños de 9 a 12 años y 20 492 adultos. Se encontraron asociaciones entre la prevalencia en niños de 9 a 12 años y la de niños de 5 a 8 años y adultos tras el tratamiento preventivo, incluso cuando solo se trató a niños en edad escolar. Cuando la prevalencia en niños de 9 a 12 años era inferior al 10%, la prevalencia en niños de 5 a 8 años era siempre inferior al 10%. Cuando la prevalencia en niños de 9 a 12 años era inferior al 50%, la prevalencia en adultos después de dos o cuatro ciclos de quimioterapia preventiva era entre un 10% y un 15% inferior que antes de la quimioterapia. Las asociaciones entre grupos de edad tras la quimioterapia fueron coherentes con las asociaciones previas a la quimioterapia en este análisis y en estudios anteriores.

**Conclusión:**

La prevalencia de la infección por *S.* *mansoni* en niños de 9 a 12 años se asoció con la prevalencia en otros grupos de edad. Además, podría utilizarse para orientar las decisiones de tratamiento en la comunidad.

## ملخص

**الغرض:**

تحديد ما إذا كان انتشار داء البلهارسيا لدى الأطفال من عمر 9 إلى 12 سنة، يرتبط بانتشاره لدى الأطفال من عمر 5 إلى 8 سنوات والبالغين بعد العلاج الكيميائي الوقائي في المدارس أو المجتمع.

**الطريقة:**

قمنا بجمع البيانات من 4 تجارب مجتمعية عشوائية للعلاج الكيميائي الوقائي في الفئات السكانية غير الخاضعة للعلاج، في كل من كوت ديفوار وكينيا وجمهورية تنزانيا المتحدة، خلال الفترة من 2010 إلى 2016 وفقًا لعدد العلاجات باستخدام البرازيكوانتيل، وطريقة تقديم العلاج. تم اكتشاف عدوى البلهارسيا المنسونية على شريحتين تم إعدادهما من البراز الأول لكل مشارك باستخدام تقنية Kato–Katz. قمنا بتقييم الارتباطات بين انتشار البلهارسيا المنسونية لدى الأطفال من عمر 9 إلى 12 سنة، والذين تتراوح أعمارهم من 5 إلى 8 سنوات والبالغين في المجتمع، قبل وبعد العلاج باستخدام نماذج التحوف Bayesian.

**النتائج:**

تم تحليل عينات البراز المأخوذة من 47985 طفلًا، وتتراوح أعمارهم من 5 إلى 8 سنوات، و81077 طفلًا، وتتراوح أعمارهم من 9 إلى 12 سنة، فضلًا عن 20492 من البالغين. لقد وجدنا ارتباطات بين معدل الانتشار لدى الأطفال من عمر 9 إلى 12 سنة، وبين الأطفال من عمر 5 إلى 8 سنوات، والبالغين بعد العلاج الوقائي، حتى عندما تم علاج الأطفال في سن المدرسة فقط. عندما كان معدل الانتشار لدى الأطفال من عمر 9 إلى 12 سنة أقل من %10، كان معدل الانتشار لدى الأطفال من 5 إلى 8 سنوات باستمرار أقل من %10. عندما كان معدل الانتشار لدى الأطفال من عمر 9 و12 سنة أقل من %50، كان معدل الانتشار لدى البالغين بعد جولتين أو أربع جولات من العلاج الكيميائي الوقائي، أقل بنسبة 10 إلى %15 عن مثيلاتها لما قبل العلاج الكيميائي. وكانت الارتباطات بين الفئات العمرية بعد العلاج الكيميائي متسقة مع هذه الارتباطات لما قبل العلاج الكيميائي في هذا التحليل والدراسات السابقة.

**الاستنتاج:**

ارتبط انتشار عدوى البلهارسيا المنسونية لدى الأطفال من عمر 9 إلى 12 سنة، بانتشارها في الفئات العمرية الأخرى ويمكن استخدامها لتوجيه قرارات العلاج المجتمعية.

## 摘要

**目的:**

确定 9 至 12 岁儿童的血吸虫病患病率是否与在学校或社区进行预防性化疗后 5 至 8 岁儿童及成人的血吸虫病患病率相关。

**方法:**

我们根据吡喹酮治疗疗程数及其给药方式，汇总了 2010 年至 2016 年间针对科特迪瓦、肯尼亚和坦桑尼亚联合共和国未接受过治疗的人群的四项社区随机预防性化疗试验数据。我们从每位参与者的第一次粪便中采样制备出两张载玻片，然后在这两张载玻片上使用改良加藤厚涂片法（Kato-Katz 技术）检测到了*曼氏血吸虫*感染。我们使用贝叶斯回归模型评估了 9 至 12 岁儿童的患病率和社区内 5 至 8 岁儿童及成人患者治疗前后*曼氏血吸虫*患病率之间的相关性**。**

**结果:**

我们分析了 47,985 名 5 至 8 岁儿童、81,077 名 9 至 12 岁儿童和 20,492 名成人的粪便样本。我们发现，即使只有学龄儿童接受了预防性治疗，9 至 12 岁儿童的患病率也与 5 至 8 岁儿童及成年人的患病率存在相关性。当 9 至 12 岁儿童的患病率低于 10% 时，5 至 8 岁儿童的患病率也一直低于 10%。当 9 至 12 岁儿童的患病率低于 50% 时，进行过两轮或四轮预防性化疗后的成人的患病率比化疗前低 10-15%。在本分析和之前的研究中，化疗后不同年龄段之前患病率的相关性与化疗前的相关性一致。

**结论:**

9 至 12 岁儿童*曼氏血吸虫*感染的患病率与其他年龄段的患病率之间存在相关性，这一结论可用于指导社区治疗决策。

## Резюме

**Цель:**

Определить, связана ли распространенность шистосомоза среди детей в возрасте 9–12 лет с распространенностью среди детей в возрасте 5–8 лет и взрослых после проведения профилактической химиотерапии в школах или общине.

**Методы:**

Данные четырех общинных рандомизированных исследований профилактической химиотерапии в популяциях, не получающих лечения, в Кении, Кот-д'Ивуаре и Объединенной Республике Танзании за 2010–2016 гг. были объединены в зависимости от количества курсов лечения празиквантелом и метода доставки. Инфекция *Schistosoma mansoni* обнаруживалась на двух предметных стеклах с образцами первого стула каждого участника с использованием метода Като – Каца. При помощи моделей байесовской линейной регрессии была проведена оценка взаимосвязей между распространенностью *S. mansoni* среди детей в возрасте 9–12 и 5–8 лет и взрослых в общине до и после лечения.

**Результаты:**

Были проанализированы образцы кала 47 985 детей в возрасте 5–8 лет, 81 077 детей в возрасте 9–12 лет и 20 492 взрослых. По результатам профилактического лечения была выявлена взаимосвязь между распространенностью заболевания у детей в возрасте 9–12 лет и у детей 5–8 лет и взрослых, даже если лечение проходили только дети школьного возраста. Если среди детей 9–12 лет распространенность заболевания составляла менее 10%, то среди детей 5–8 лет она постоянно была ниже 10%. Если распространенность заболевания среди детей в возрасте 9–12 лет составляла менее 50%, то среди взрослых после двух или четырех раундов профилактической химиотерапии распространенность заболевания была на 10–15% ниже, чем до химиотерапии. В данном анализе и предыдущих исследованиях взаимосвязь возрастных групп после химиотерапии соответствовала взаимосвязи до химиотерапии.

**Вывод:**

Распространенность инфекции *S. mansoni* среди детей в возрасте 9–12 лет была связана с распространенностью в других возрастных группах и может быть использована для принятия решений о лечении в общинах.

## Introduction

Control programmes for schistosomiasis, the human disease caused by worms of the genus *Schistosoma*, focus on collecting and evaluating data from school-age children.^1^ The use of school-age children as a sentinel population is considered cost-effective for neglected tropical disease programmes because:^1^^–^^5^ (i) these children typically have the highest infection prevalence;^1^ (ii) their burden-of-disease indicators are assumed to be representative of the community;^6^ and (iii) preventive chemotherapy is mainly disseminated through schools due to the lower overall cost and ease of distribution.^7^

To ensure the control and elimination of schistosomiasis are sustained, other age groups should be also covered by control measures.^7^^–^^9^ The 2022 human schistosomiasis guideline from the World Health Organization (WHO) recommended that everyone over the age of 2 years should be treated.^10^ However, monitoring different age groups necessitates collecting data from these age groups to guide accurate drug delivery at a time when funds for monitoring and evaluation are already insufficient. There are also logistic challenges in collecting parasitological data from multiple age groups, especially among adults,^4^ and expanding the age range for monitoring and evaluation will likely increase costs and delay reporting.

Prior research found associations between various community-level parasitological indicators in different age groups in western Kenya before praziquantel dissemination.^4^ Our study aimed to extend this observation by examining whether the prevalence of schistosomiasis in children aged 9–12 years is associated with the relative prevalence in other age groups after mass treatment and to determine whether the delivery of treatment in only schools or throughout the community affects any associations. Specifically, we wanted to determine if the assessment of stool samples from children aged 9–12 years could be used to monitor the preventive chemotherapy target of 10% infection prevalence for other age groups.^10^ To do this, we pooled data from four community-level, praziquantel-dissemination trials collected both before the initiation of preventive chemotherapy and after 4 years of the intervention. These four trials were conducted under a unified protocol, which provided a unique opportunity to combine data and to evaluate correlations between different age groups.^11^

Our secondary objective was to determine whether the impact of preventive chemotherapy was influenced by the delivery method. We hypothesized that community-wide, preventive chemotherapy would have a similar effect in all age groups, whereas school-based treatment would disproportionately decrease the prevalence in children aged 9–12 years, which would make the prevalence in this age group after the administration of preventive chemotherapy less useful as a proxy for the prevalence in younger children or adults. However, because we previously observed that the prevalence in adults decreased even in communities where treatment was delivered only in schools,^12^ we also tested the hypothesis that the prevalence in children aged 9–12 years could serve as a predictor of the relative prevalence in children aged 5–8 years and in adults.

## Methods

The data used in our analysis were pooled from four community-randomized, preventive, chemotherapy interventions organized by the Schistosomiasis Consortium for Operational Research and Evaluation (SCORE) under a unified protocol that were conducted between 2010 and 2016.^11^ Two trials, one in Côte d’Ivoire and one in western Kenya,^13^^,^^14^ involved treatment-naïve communities that were selected because the prevalence of *Schistosoma mansoni* infection in schoolchildren aged 13–14 years was 10%–24%. The remaining two trials, one in western Kenya and one in the United Republic of Tanzania,^12^^,^^15^ involved treatment-naïve communities selected because the *S. mansoni* infection prevalence was 25% or more in schoolchildren aged 13–14 years. All data were publicly available and were downloaded from ClinEpiDB.^16^

**Fig. 1:**
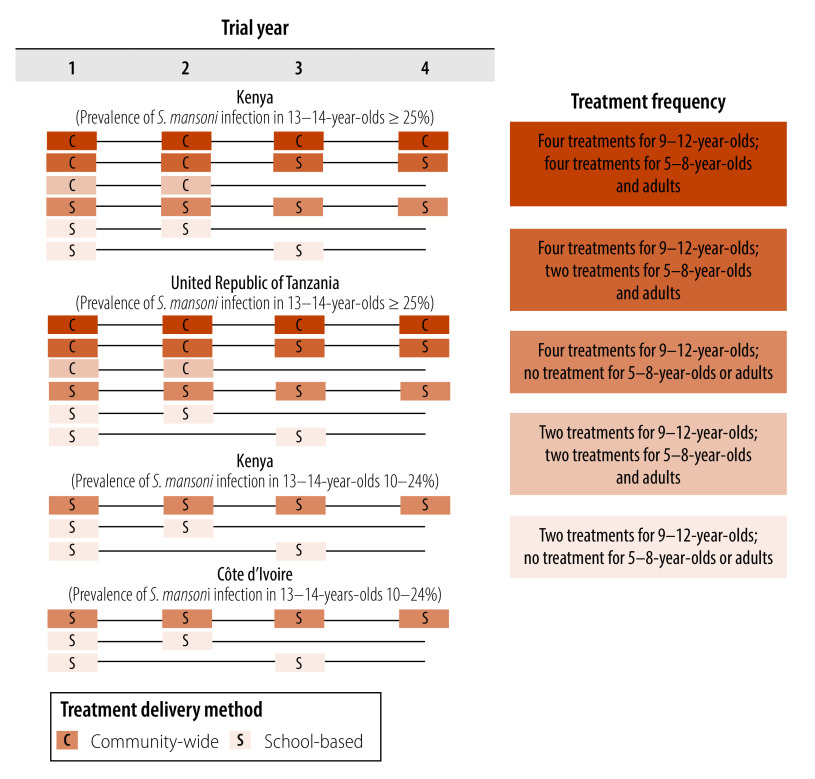
Praziquantel distribution schedules in four trials, study of age-group associations of schistosomiasis prevalence, Côte d’Ivoire, Kenya and United Republic of Tanzania, 2010–2016

A thorough description of the trial methods is included in the online repository.^17^ In brief, communities in each trial were randomized to different annual praziquantel distribution schedules for 2 or 4 years (Fig. 1). Data were collected from children aged 5–8 years, children aged 9–12 years and adults aged 20–55 years at both baseline (i.e. before treatment in year 1) and in year 5 (the non-treatment year), 1 year after preventive chemotherapy was last administered. Being naïve to preventative chemotherapy, children aged 5–8 years could serve as a proxy for younger children (e.g. pre-school-age children). Treatment coverage of children aged 9–12 years was above 70% in most trial arms.^12^^–^^15^ Data from communities in the four trials that had the same number and type (i.e. community-wide or school-based) of treatment were pooled and analysed separately (Fig. 1). With community-wide-treatment, all age groups were considered treated; with school-based-treatment, only children aged 9–12 years were considered treated. Our analysis included five treatment frequencies for 9–12-year-olds and 5–8-year-olds: (i) four treatments for both 9–12-year-olds and 5–8-year-olds; (ii) four treatments for 9–12-year-olds and two treatments for 5–8-year-olds; (iii) four treatments for 9–12-year-olds and no treatment for 5–8-year-olds; (iv) two treatments for both 9–12-year-olds and 5–8-year-olds; and (v) two treatments for 9–12-year-olds and no treatment for 5–8-year-olds. The analysis also included similar treatment frequencies for children aged 9–12 years and adults.

*S. mansoni: Schistosoma mansoni.*

In years when a community was scheduled for treatment, whether community-wide or school-based, the prevalence and intensity of *S. mansoni* infection were measured before the distribution of praziquantel. In all trial arms, attempts were made to collect three stool samples from approximately 100 children aged 9–12 years in each treatment year (parasitological indicators were not assessed in non-treatment years) and in year 5, 1 year after the last round of treatment. A single stool sample was collected in year 1 and year 5 from at most 100 children aged 5–8 years and from 50 adults. Samples were obtained from children aged 5–8 years, irrespective of whether the baseline prevalence in schoolchildren aged 13–14 years was 10%–24% or 25% or higher. Samples from adults were collected only in communities where the baseline prevalence in schoolchildren aged 13–14 years was 25% or higher.

Two slides were prepared from each stool sample using the Kato–Katz thick smear technique. Each slide was examined by a trained microscopist who reported the number of parasite eggs per slide. For our analysis, we excluded participants who did not have two slides available from a single stool sample. For 9–12-year-olds, our analysis used findings from the first stool sample for which two slides were available. A participant was considered positive for *S. mansoni* infection if eggs were detected on either slide.

### Statistical methods

We assessed associations between prevalence estimates for different age groups using a binomial, Bayesian errors-in-variables model.^18^^,^^19^ Models were fitted by Markov chain Monte Carlo methods using the rjags package in R (R Foundation, Vienna, Austria).^20^ We created fitted curves for year 1 and year 5 for the estimated association between the community-level prevalence of *S. mansoni* infection in 9–12-year-olds and the community-level prevalence in other age groups. Further details of the statistical analyses and the model selection can be found in the online repository.^17^

We performed additional analyses using data collected at year 5 to evaluate associations between the infection prevalence in 9–12-year-olds and the prevalence in other age groups for different treatment frequencies and dissemination methods (i.e. school-based and community-wide). As the best-fitting model in most situations was a linear model (on the log scale), we fitted two models: one for 5–8-year-olds and one for adults. Each model included: (i) the prevalence in 9–12-year-olds as an errors-in-variables term; (ii) variables for the treatment frequency and treatment schedule; and (iii) interactions between the prevalence in 9–12-year-olds and the treatment schedule. The relative likelihood that a 5–8-year-old in a particular community would have an *S. mansoni* infection compared with a 5–8-year-old in a community where the infection prevalence in 9–12-year-olds was 10 percentage points higher is reported as an odds ratio (with a 95% Bayesian credible interval; BCI).

All analyses were conducted using R version 4.0.3. Information on ethical approval can be found in the online repository and elsewhere.^12^^–^^15^^,^^17^

## Results

Stool samples from 47 985 children aged 5–8 years, 81 077 children aged 9–12 years and 20 492 adults were analysed. Data were available from 49 to 50 communities to compare the prevalence of *S. mansoni* infection in 5–8-year-olds and 9–12-year-olds in year 1 and year 5 for three of the five treatment groups shown in Fig. 1 (Table 1). For the treatment group in which 9–12-year-olds received four treatments and 5–8-year-olds received none, data were available from 100 communities for both years. For the treatment group in which 9–12-year-olds received two treatments and 5–8-year-olds received none, data were available from 200 communities for both years. Although the estimated infection prevalence in participants and communities decreased from year 1 to year 5 across all treatment groups, decreases were smaller when 5–8-year-olds were not treated during the study, possibly because the prevalence in year 1 was low for both 5–8-year-olds and 9–12-year-olds in these treatment groups.

**Table 1:** Children aged 5–8 years and 9–12 years, study of age-group associations of schistosomiasis prevalence, Côte d’Ivoire, Kenya and United Republic of Tanzania, 2010–2016

No. praziquantel treatments received,^a^ 5–8-year-olds: 9–12-year-olds	Trial year	No. communities in trials	Individual parameters						Community parameters, median range				
			5–8-year-olds			9–12-year-olds			5–8-year-olds			9–12-year-olds	
			No. participants in trials	Prevalence of *S. mansoni* infection, % (95% CI)		No. participants in trials	Prevalence of *S. mansoni* infection, % (95% CI)		No. participants per community	Prevalence of *S. mansoni* infection in communities, %		No. participants per community	Prevalence of *S. mansoni* infection in communities, %
0:2	1	200	10 323	24.0 (19.9–28.6)		16 946	29.9 (26.5–33.6)		43 (3–141)	10.3 (0.0–100)		96 (27–123)	18.9 (0.0–100)
0:2	5	200	10 753	20.3 (16.4–24.7)		19 193	19.0 (16.1–22.1)		47 (6–108)	6.5 (0.0–93.1)		100 (36–127)	10.0 (0.0–93.0)
2:2	1	49	2 587	35.1 (26.2–44.9)		4 348	42.4 (34.6–50.4)		45 (10–100)	25.9 (0.0–88.0)		98 (33–110)	41.0 (7.0–97.0)
2:2	5	50	2 977	25.7 (18.6–33.7)		4 820	26.2 (20.3–32.9)		51 (14–120)	13.2 (0.0–87.5)		100 (67–102)	19.2 (0.0–89.0)
0:4	1	100	4 588	21.4 (15.7–28.2)		8 260	27.2 (22.2–32.7)		38 (3–100)	7.7 (0.0–95.7)		96 (12–120)	15.2 (0.0–89.0)
0:4	5	100	4 962	18.9 (13.6–25.2)		9 500	15.0 (11.3–19.4)		42 (8–109)	4.5 (0.0–79.4)		100 (40–130)	6.0 (0.0–79.0)
2:4	1	49	2 717	32.4 (24.4–41.3)		4 344	40.0 (32.1–48.4)		44 (7–105)	15.4 (0.0–100)		97 (11–120)	27.7 (3.0–94.0)
2:4	5	50	2 968	26.4 (18.7–35.2)		4 755	22.2 (15.6–29.9)		55 (9–109)	12.5 (0.0–83.0)		100 (51–120)	10.0 (0.0–84.0)
4:4	1	49	3 089	42.5 (33.4–51.9)		4 248	46.7 (39.1–54.4)		65 (7–152)	34.4 (0.0–100)		99 (34–111)	44.6 (3.7–92.3)
4:4	5	49	2 958	23.6 (17.8–30.3)		4 652	23.8 (17.7–30.8)		59 (11–101)	13.3 (0.0–71.0)		100 (59–109)	11.9 (1.0–77.8)

CI: confidence interval; S. mansoni: *Schistosoma mansoni*.

^a^ Praziquantel was administered annually in years 1 to 4, as indicated.

Data were available from 40 to 50 communities to compare the infection prevalence in adults and 9–12-year-olds for four of the five treatment groups (Table 2). For the treatment group in which 9–12-year-olds received two treatments and adults received none, data were available from 98 communities for year 1 and from 87 communities for year 5.

**Table 2:** Adults and children aged 9–12 years, study of age-group associations of schistosomiasis prevalence, Côte d’Ivoire, Kenya and United Republic of Tanzania, 2010–2016

No. praziquantel treatments received,^a^ adults^b^: 9–12-year-olds	Trial year	No. communities in trials	Individual parameters						Community parameters, median (range)				
			Adults^b^			9–12-year-olds			Adults^b^			9–12-year-olds	
			No. participants in trials	Prevalence of *S. mansoni* infection, % (95% CI)		No. participants in trials	Prevalence of *S. mansoni* infection, % (95% CI)		No. participants per community	Prevalence of *S. mansoni* infection in communities, %		No. participants per community	Prevalence of *S. mansoni* infection in communities, %
0:2	1	98	4064	38.0 (33.8–42.2)		8686	46.3 (40.9–51.9)		47 (2–54)	32.7 (0.0–87.0)		96 (27–123)	18.9 (0.0–100)
0:2	5	87	2865	18.6 (15.3–22.2)		8394	28.3 (23.0–34.1)		43 (1–79)	15.9 (0.0–82.0)		100 (36–127)	10.0 (0.0–93.0)
2:2	1	48	1876	36.1 (31.4–41.1)		4240	42.9 (35.0–51.1)		45 (6–53)	32.0 (0.0–76.2)		98 (33–110)	41.0 (7.0–97.0)
2:2	5	44	1387	15.3 (11.6–19.7)		4232	25.2 (18.7–32.6)		36 (1–50)	14.3 (0.0–100)		100 (67–102)	19.2 (0.0–89.0)
0:4	1	48	1926	36.2 (30.3–42.5)		4137	43.1 (35.2–51.3)		47 (6–60)	31.2 (0.0–78.0)		96 (12–120)	15.2 (0.0–89.0)
0:4	5	41	1364	17.7 (12.4–24.1)		3709	24.9 (17.8–33.1)		43 (1–50)	12.5 (0.0–56.5)		100 (40–130)	6.0 (0.0–79.0)
2:4	1	50	2096	35.4 (29.4–41.7)		4355	40.0 (32.0–48.3)		48 (4–54)	32.3 (5.1–88.4)		96.5 (11–120)	27.7 (3.0–94.0)
2:4	5	44	1413	14.1 (9.7–19.5)		4155	18.3 (12.3–25.7)		45 (1–50)	13.0 (0.0–100)		100 (51–120)	10.0 (0.0–84.0)
4:4	1	49	1983	40.3 (35.3–45.5)		4248	46.7 (39.1–54.4)		48 (7–51)	41.7 (0.0–78.7)		99 (34–111)	44.6 (3.7–92.3)
4:4	5	42	1468	13.6 (8.3–20.6)		3968	21.8 (15.7–28.9)		46 (1–74)	6.0 (0.0–71.4)		100 (59–109)	11.9 (1.0–77.8)

CI: confidence interval; S. mansoni: *Schistosoma mansoni*.

^a^ Praziquantel was administered annually in years 1 to 4, as indicated.

^b^ Adults were aged between 20 and 55 years.

**Fig. 2:**
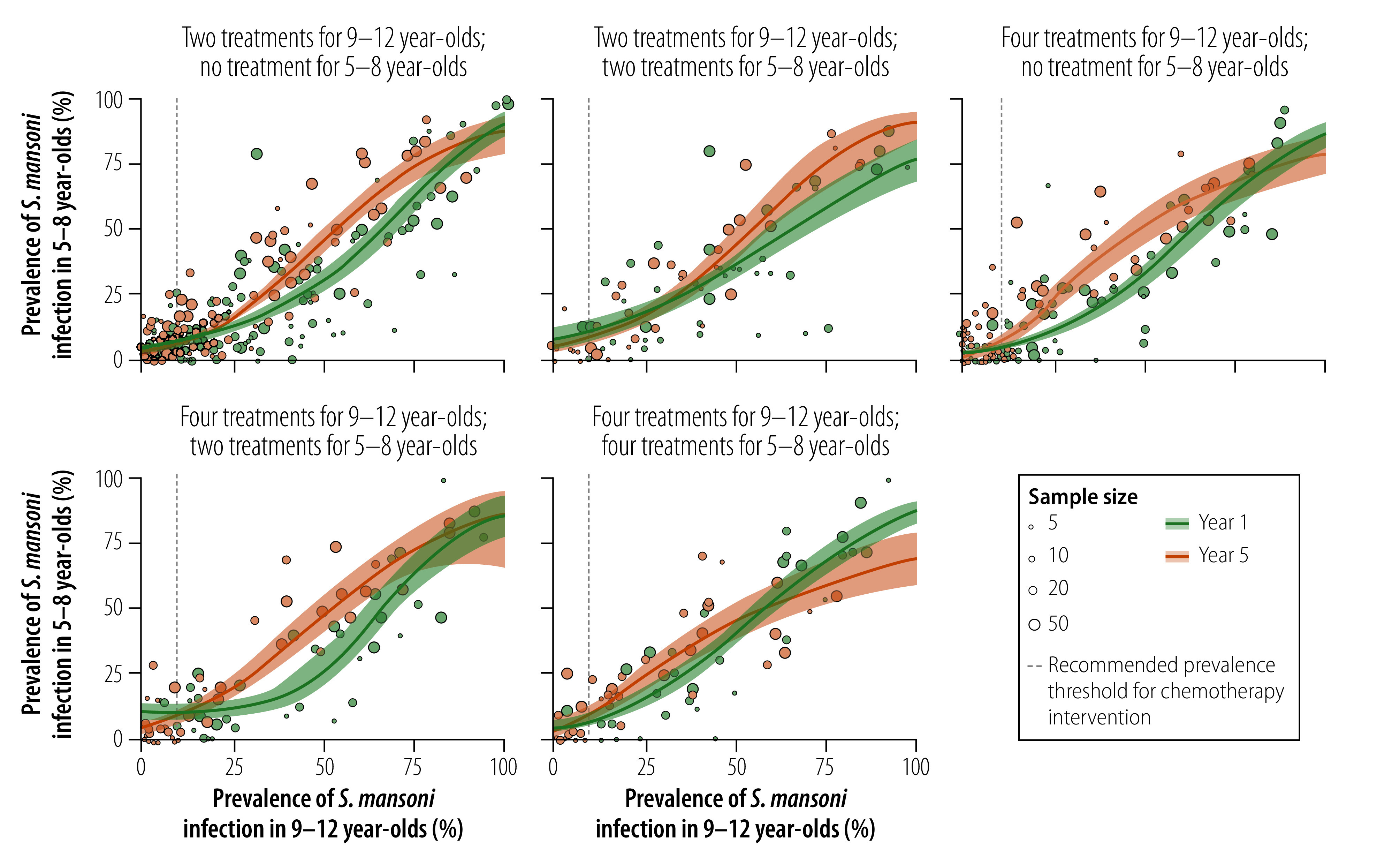
Associations between the community-level prevalence of *Schistosoma mansoni* infection in 9–12-year-olds and 5–8-year-olds, study of age-group associations of schistosomiasis prevalence, Côte d’Ivoire, Kenya and United Republic of Tanzania, 2010–2016

Associations between the prevalence of *S. mansoni* infection in 9–12-year-olds and 5–8-year-olds were roughly similar for the five treatment frequencies (Fig. 2). When the prevalence in 9–12-year-olds was less than 10%, the difference in prevalence between year 1 and year 5 in 5–8-year-olds was less than 3.4 percentage points for all treatment frequencies except that with which 9–12-year-olds received four treatments and 5–8-year-olds received two treatments, where the association graph was more curvilinear. Generally, when the prevalence in 9–12-year-olds was between 25% and 50%, the prevalence in 5–8-year-olds was lower in year 1 than year 5. Fitted curves for the two years tended to converge again when the prevalence in 9–12-year-olds was 60% to 90%, except for the treatment frequency where both 9–12-year-olds and 5–8-year-olds received two treatments. However, as the prevalence in 9–12-year-olds was greater than 75% in very few communities, especially in year 5, findings in communities in which the prevalence in 9–12-year-olds was greater than 75% should be interpreted with caution.

*S. mansoni: Schistosoma mansoni.*

Notes: The fitted curves and scatter plots show the associations between the prevalence of *S. mansoni* infection in 9–12-year-olds and 5–8-year-olds in the community before praziquantel treatment (year 1) and in year 5 after treatment for five treatment frequencies, as derived from four community-randomized, preventive chemotherapy intervention trials. Praziquantel treatment lasted for up to 4 years.

**Fig. 3:**
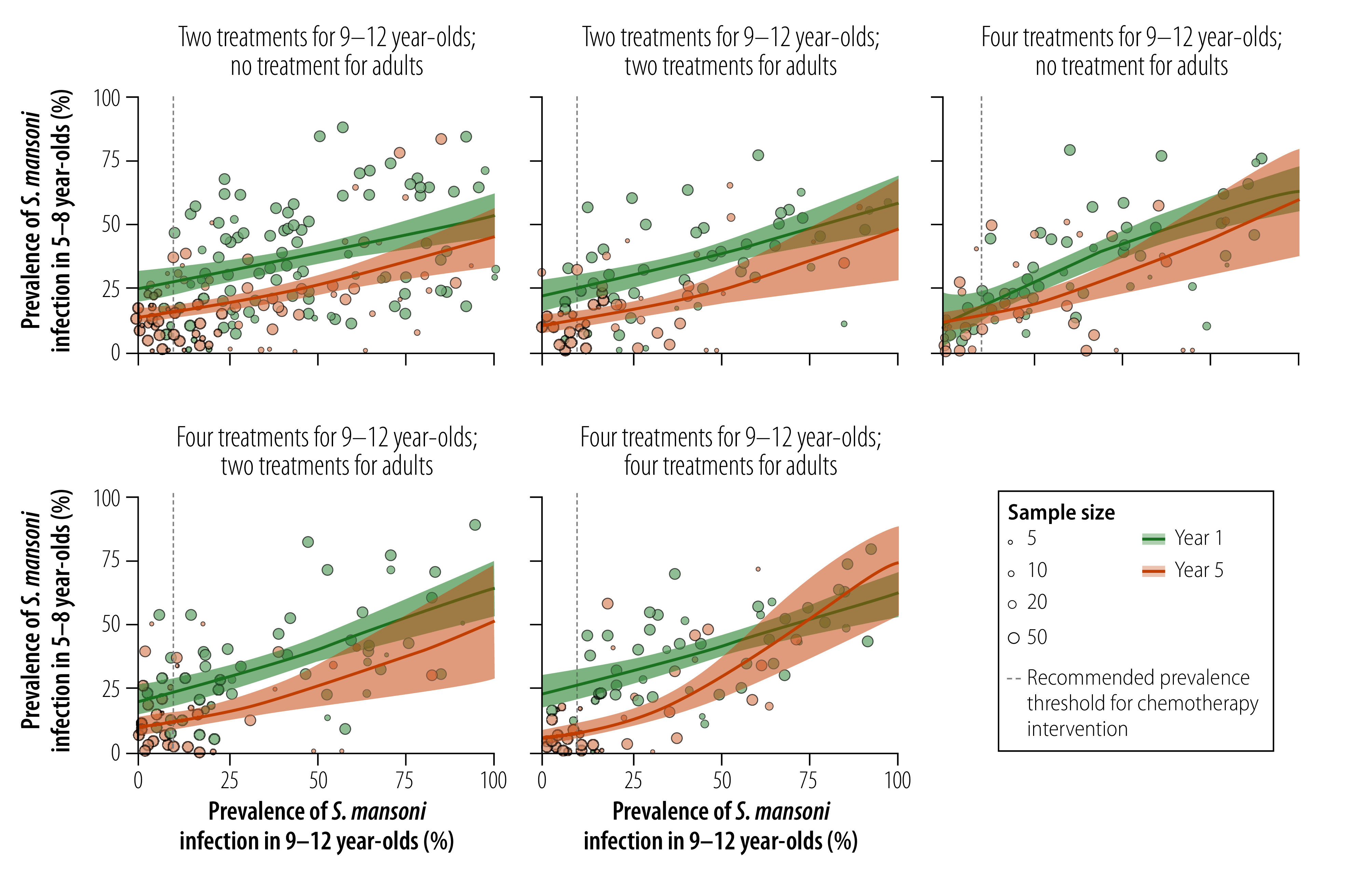
Associations between the community-level prevalence of *Schistosoma mansoni* infection in 9–12-year-olds and adults, study of age-group associations of schistosomiasis prevalence, Côte d’Ivoire, Kenya and United Republic of Tanzania, 2010–2016

Fig. 3 shows associations between the prevalence of *S. mansoni* infection in 9–12-year-olds and adults for the five treatment frequencies. When the prevalence in 9–12-year-olds was less than 50%, the association between the prevalence in 9–12-year-olds and that in adults differed markedly between year 1 and year 5. For instance, for the treatment frequency where 9–12-year-olds were treated twice and adults were not treated, when the prevalence in 9–12-year-olds was less than 25%, the prevalence in adults in year 5 was consistently lower than the prevalence in adults in year 1 (difference range: 11.5–12.4 percentage points). The same was true for the two treatment frequencies where 9–12-year-olds were treated four times and adults were treated two or four times, respectively. For the treatment frequency in which 9–12-year-olds were treated four times and adults were not treated, the difference in prevalence in adults between year 1 and year 5 was much smaller and more variable (range: 0.1–8.0 percentage points) due to the curvilinear shape of the year-1 curve. Similar to our observations on the associations between the prevalence in 9–12-year-olds and 5–8-year-olds, the difference in prevalence between year 1 and year 5 in adults became less pronounced when the prevalence in 9–12-year-olds increased above 50%. Again, this result should be interpreted with caution.

*S. mansoni: Schistosoma mansoni.*

Notes: The fitted curves and scatter plots show the associations between the prevalence of *S. mansoni* infection in 9–12-year-olds and adults in the community before praziquantel treatment (year 1) and in year 5 after treatment for five treatment frequencies, as derived from four community-randomized, preventive chemotherapy intervention trials. Adults were aged between 20 and 55 years. Praziquantel treatment lasted for up to 4 years.

Associations between the prevalence of *S. mansoni* infection in 9–12-year-olds and that in 5–8-year-olds at year 5 were robust and similar for all five treatment frequencies (Table 3). Based on the associations found, the odds that a 5–8-year-old in a particular community would test positive on a Kato–Katz stool examination compared with a 5–8-year-old in a community where the infection prevalence in 9–12-year-olds was 10 percentage points higher would be between 0.54 (BCI: 0.50–0.58) and 0.64 (BCI: 0.58–0.70), depending on the treatment frequency and dissemination method. The BCIs for the odds ratios generally overlapped for different treatment frequencies and dissemination methods, which suggests that the association between the prevalence in 5–8-year-olds and 9–12-year-olds at year 5 was similar for different treatment frequencies and dissemination methods.

**Table 3:** Association between *Schistosoma mansoni* prevalence in children aged 9–12 years and 5–8 years, study of age-group associations of schistosomiasis prevalence, Côte d’Ivoire, Kenya and United Republic of Tanzania, 2010–2016

No. praziquantel treatments received, 9–12-year-olds:5–8-year-olds	Treatment dissemination method	Relative likelihood of *S. mansoni* infection in 5–8-year-olds for a 10% drop in the infection rate in 9–12-year-olds OR (95% BCI)^a^
2:0	School-based	0.56 (0.53–0.59)
2:2	Community-wide	0.58 (0.52–0.64)
4:0	School-based	0.54 (0.50–0.58)
4:2	Community-wide and school-based	0.59 (0.54–0.64)
4:4	Community-wide	0.64 (0.58–0.70)

BCI: Bayesian credible interval; OR: odds ratio; *S. mansoni: Schistosoma mansoni*.

^a^ The OR expresses the relative likelihood that a 5–8-year-old in a particular community would have an *S. mansoni* infection compared with a 5–8-year-old in a community where the infection prevalence in 9–12-year-olds was 10 percentage points higher. For example, in the treatment group where 9–12-year-olds received two treatments and 5–8-year-olds received none, a 10% decrease in the *S. mansoni* infection rate among 9–12-year-olds would correspond to a reduced likelihood of infection among 5–8-year-olds, expressed as an odds ratio of 0.56.

Associations between the prevalence of *S. mansoni* infection in 9–12-year-olds and that in adults at year 5 were weaker and more variable than analogous associations between the prevalence in 9–12-year-olds and 5–8-year-olds (Table 4). On comparing a community where the prevalence in 9–12-year-olds was 10 percentage points lower than in another community, the odds that an adult in the first community would test positive on a Kato–Katz stool examination compared with an adult in the second community would be between 0.67 (BCI: 0.58–0.77) and 0.84 (BCI: 0.79–0.90), depending on the treatment schedule and dissemination method. Although BCIs for the odds ratios generally overlapped for different treatment frequencies and dissemination methods, there was a weak trend for the odds of a positive examination result to decrease as the number of treatments increased.

**Table 4:** Association between *Schistosoma mansoni* prevalence in children aged 9–12 years and adults, study of age-group associations of schistosomiasis prevalence, Côte d’Ivoire, Kenya and United Republic of Tanzania, 2010–2016

No. praziquantel treatments received, 9–12-year-olds:adults^b^	Treatment dissemination method	Relative likelihood of an *S. mansoni* infection in an adult for a 10% drop in infection rate in 9–12-year-olds OR (95% BCI)^a^
2:0	School-based	0.84 (0.79–0.90)
2:2	Community-wide	0.81 (0.72–0.90)
4:0	School-based	0.77 (0.69–0.87)
4:2	Community-wide and school-based	0.79 (0.70–0.89)
4:4	Community-wide	0.67 (0.58–0.77)

BCI: Bayesian credible interval; OR: odds ratio; *S. mansoni: Schistosoma mansoni*.

^a^ The OR expresses the relative likelihood that an adult in a particular community would have an *S. mansoni* infection compared with an adult in a community where the infection prevalence in 9–12-year-olds was 10 percentage points higher. For example, in the treatment group where 9–12-year-olds received two treatments and adults received none, a 10% decrease in the *S. mansoni* infection rate among 9–12-year-olds would correspond to a reduced likelihood of infection among adults, expressed as an odds ratio of 0.84.

^b^ Adults were aged between 20 and 55 years.

## Discussion

Our findings suggest that measurements of the prevalence of *S. mansoni* infection in stool samples from 9–12-year-olds during schistosomiasis control programmes can be used to monitor the relative prevalence in other age groups, both before and after two to four rounds of preventive chemotherapy. Furthermore, we found that once the prevalence in children aged 9–12 years is reduced to below 10%, the prevalence in 5–8-year-olds should also be less than 10%. Thus, the prevalence in 9–12-year-olds could provide an upper bound for the prevalence in 5–8-year-olds, potentially eliminating the need to monitor and evaluate 5–8-year-olds and, by proxy, pre-school-age children. As the recent WHO schistosomiasis guideline uses 10% as a prevalence target for control programmes,^10^ it may be reasonable to expect that once a programme has achieved a prevalence of 10% or less among children aged 9–12 years, the prevalence in younger children will also be below 10%.

Although it seems likely that the prevalence of *S. mansoni* infection in adults will be below 10% when the prevalence in 9–12-year-olds is below 10%, further research is needed. We found that the prevalence in adults decreased more between year 1 and year 5 in the community trials we analysed than the prevalence in 9–12-year-olds. The Secor et al. trial in Kenya in our study and the SCORE *S. haematobium* preventive chemotherapy trial both reported a decrease in the prevalence in adults,^12^^,^^21^ even in communities that received only school-based treatment. The researchers attributed the decrease to reduced community-wide transmission due to preventive chemotherapy. However, the difference in model-based estimates between year 1 and year 5 we observed make it difficult to use the prevalence in 9–12-year-olds as a proxy for the prevalence in adults: using the prevalence in 9–12-year-olds in year 1 to estimate the prevalence in adults would result in an estimate 10% to 15% higher than using the prevalence in 9–12-year-olds in year 5. Nevertheless, as the prevalence in adults was observed to increase consistently with the increase in prevalence in 9–12-year-olds in both year 1 and year 5, it is likely that the prevalence in adults can also be predicted by the prevalence in 9–12-year-olds once further research has established the appropriate association.

If the relationship between the prevalence of *S. mansoni* infection in children aged 9–12 years and that in other age groups can be established, monitoring and evaluating 9–12-year-olds would be sufficient to provide estimates of the prevalence in other age groups. This approach would reduce the financial burden on control programmes as data collection could focus on primary schools. Further groundwork is needed, however. First, our analyses compared the infection prevalence before and after preventive chemotherapy. Researchers have shown that the association between the prevalence in 9–12-year-olds and other age groups can change rapidly in the first few years after a preventive chemotherapy programme.^12^ Moreover, changes in prevalence at the community level may differ between age groups because of differences in exposure to infection and in immunity,^22^ though the pattern may alter after several years of treatment as the annual reduction in prevalence would be expected to decrease. Second, the relationship between the prevalence in 9–12-year-olds and that in other age groups may vary by location. Associations between age groups may need to be established for each geographical setting because spatial correlations between prevalence values have been recognized as important for disease elimination and the delivery of preventive chemotherapy.^23^^,^^24^ Data sources with schistosome prevalence data for different age groups could be used to further evaluate the relationship between the prevalence in children aged 9–12 years and that in other age groups.

Our results differ from those of a modelling study,^9^ which concluded that adult data should also be collected. In that study, preventive chemotherapy was required for adults to achieve disease control and elimination in regions where the infection burden in adults was high. In general, assumptions made about the initial prevalence in modelling studies have differed from our observations.^9^^,^^25^^–^^28^ For example, all modelling scenarios considered assumed that the prevalence of infection in adults at treatment initiation was above 60.0%.^9^ In the treatment-naïve areas included in our study, the prevalence of *S. mansoni* infection in adults was no higher than 40.3% for any treatment group. Furthermore, all modelling scenarios assumed that the prevalence in school-age children was 75% at treatment initiation, whereas we found an infection prevalence no higher than 46.7% in 9–12-year-olds in any treatment group in the trials included. However, the data we used were based on evaluations of single stool samples and were likely to have been underestimates. Finally, the decreases in prevalence we observed in 9–12-year-olds and adults were larger than those predicted in the modelling scenarios.^9^ In the trials in our analysis, the infection prevalence in 9–12-year-olds decreased by approximately half over the study period; the mathematical models assumed that the prevalence in school-age children would drop by no more than one third over 5 years, and that the prevalence in adults would remain largely unchanged. If these models were updated to incorporate the lower prevalence rates from the trials in our study, the modelling predictions may agree with our conclusions.

One shortcoming of our analysis is that, in monitoring younger children, there is generally more of a focus on pre-school-age children than on 5–8-year-olds. Pre-school-age children were not included in the trials in our study because they are not currently involved in regular preventive chemotherapy campaigns. Nevertheless, we hope that our findings in 5–8-year-olds can provide some understanding of the relationship between the prevalence of *S. mansoni* infection in pre-school-age children and the prevalence in 9–12-year-olds at the community level because the 5–8-year-olds were treatment-naïve. The prevalence in younger children is expected to be lower than in children in their first year of school because they will have had fewer years of possible exposure to infection. More data are needed to explore the relationship between the infection prevalence in pre-school-age children and 9–12-year-olds at the community level.

Our results may also have been affected by biases associated with other shortcomings. First, the trials included used only one stool sample from which two slides were obtained. This approach enabled the prevalence of *S. mansoni* infection to be compared between different age groups, as the sensitivity of the test would be expected to be the same in each group. However, the prevalence was likely to have been underestimated because the sensitivity of the Kato–Katz technique is low when only a single stool is evaluated.^29^^,^^30^ Second, as our data came from community-randomized trials, our findings may not be generalizable to control programmes. Third, as the trials used the infection prevalence in 13–14-year-old schoolchildren as an eligibility criterion for communities, some communities in which the prevalence in 9–12-year-olds was less than 25% may have been included in the analysis along with trials in communities where the prevalence was 25% or more. Third, although the data came from multiple locations, they do not represent a complete set of schistosomiasis transmission archetypes. The trial sites either bordered one of the African Great Lakes (i.e. in Kenya and the United Republic of Tanzania) or were in the mountains (i.e. in Côte d’Ivoire). There were no sites bordering rivers (e.g. in the Senegal River Basin) or outside Africa (e.g. in the Americas, Asia or the Western Pacific). Associations between age groups in these other areas should be investigated because these associations may be affected by variations in ecology, local behaviour or *Schistosoma* host species. Fourth, age-group associations may also have been affected by variations in treatment coverage. Finally, as our analyses involved data on *S. mansoni*, the associations we found may not extend to other *Schistosoma* species.

In conclusion, we found that the prevalence of *S. mansoni* infection in 9–12-year-olds reflected the relative prevalence in other age groups, both before and after preventive chemotherapy, which suggests that infection monitoring in this age group is sufficient to determine the approximate prevalence in other age groups and to guide community treatment decisions. Importantly, we observed that, when a treatment threshold of 10% prevalence had been achieved in 9–12-year-olds, the prevalence in younger children was reliably lower. The relationship between the prevalence in 9–12-year-olds and adults is likely to be similar, but further studies in other locations are needed for confirmation.
